# Active Carbon Respiratory Masks as the Adsorbent of Toxic Gases in Ambient Air

**DOI:** 10.1155/2019/5283971

**Published:** 2019-06-02

**Authors:** Khayan Khayan, Taufik Anwar, Slamet Wardoyo, Widyana Lakshmi Puspita

**Affiliations:** ^1^Department of Environmental Health, Poltekkes Kemenkes Pontianak, Indonesia; ^2^Department of Nutrition, Poltekkes Kemenkes Pontianak, Indonesia

## Abstract

The air quality that is increasingly carrying out pollution as a result of pollution by human activities is of concern to the world, both developed and developing countries. The impact of air pollution is unavoidable, especially for health. Several efforts have been made to suppress the occurrence of pollution starting from the control of sources, media, and protective efforts in human beings. Focusing on protective efforts, this study was carried out by designed respiratory masks capable of adsorbing toxic gases in ambient air by utilizing mask materials on the market with the addition of activated carbon; the study was carried out with an experimental approach. Testing distinguishes the ability of cotton, spunbond, meltblown, and activated carbon as a respiratory mask to absorb toxic gases such as COx, NOx, and SOx. Test statistics are using the ANOVA test with a confidence level of 95%, *α* = 5%. The results show that combining activated carbon, spunbond, and meltblown is more effective compared to respiratory masks made from spunbond and meltblown (surgical masks) in absorbing toxic gases.

## 1. Introduction

The impact of air pollution on public health, especially high-risk populations such as children and the elderly, is still the world's attention in both developed and developing countries [1, 2]. Air pollution is still a substantial problem. For this reason, several countries have begun efforts to reduce emissions from certain sources; for example, lead has been removed from the level of gasoline and sulfur in controlled fuels [3]. Even though this action has been taken, the ambient air pollution is still high. As in tropical countries air pollution is one of them triggered by peat fires [4].

Community health problems that are still high are caused by pollution of the physical environment, water, soil, food, and air such as pollutants of heavy metals Pb, Hg, Cd, and toxic gases COx, NOx, and SOx [5, 6].

The NOx effect causes pyrolysis, convulsive nerve, and pulmonary edema. A dose of > 800 ppm results in death. COx, especially CO concentration of 100 ppm, causes health problems. COx has the ability to react 200 times faster with hemoglobin (Hb) to form COHb compared to the affinity of O2 with Hb (HbO) [7]. COHb concentration> 1% in the blood can give abnormal appearance and ≥5% changes in heart and pulmonary function. SOx levels of 8 ppm can cause throat irritation, eye irritation, and coughing. The main effects of exposure to SOx, NOx, and COx, especially in vulnerable groups, namely, elderly people and children, can cause respiratory and cardiovascular diseases [7, 8].

To reduce exposure to toxic gases in the air, as with the masses in West Kalimantan, Indonesia, the community uses several types of respiratory protection such as cotton cloth, surgical masks, and N95 respirator masks [9, 10], based on the purpose of making surgical masks in the area to protect the wearer. While the N95 respirator mask is designed to protect the wearer from the environment, surgical masks and N95 masks are designed to filter viruses and bacteria and dust [11, 12]. All types of respiratory protection have not been able to filter and absorb exposure to toxic gases such as COx, NOx, and SOx [10, 11, 13]. To overcome this, it is necessary to change the respiratory protection (face mask) which has more ability, which is able to filter bacteria, dust, and gas at once. The material is made from facial ingredients, namely, spunbond and meltblown by combining activated carbon. Activated carbon has a relatively large micropore and mesopore, so it has a large surface area. It expected to absorb toxic gases such as COx, NOx, and SOx [14, 15].

## 2. Material and Method

### 2.1. Masker Combination

Facial masks (breathing masks) are made of materials consisting of spunbond, meltblown, and activated carbon. Activated carbon is used in a granular form with dust content below 10% according to the activated carbon standard required by the Indonesian National Standard. Activated carbon is applied between spunbond and meltblown.

### 2.2. Parameter Selection

The parameters used as toxic gas indicators are COx, SOx, and NOx. The experiment was carried out on the Pontianak, West Kalimantan highway, in seven points with high motor vehicle density. The study began at 7:00 a.m. from 3:00 p.m. Western Indonesia Time. The exposure of mask material is carried out in areas that have high toxic gas contamination and a lot of people's activities.

Material masks are simulated and exposed to toxic gases (COx, SOx, and NOx) using the aid of gas suckers at the same speed as humans. The flow rate used uses the flow rate for heavy workers which is 85 L/min.

Air suction aids use Gas Sampler Ambient (DEKKO 642 N, Japan), which is designed in such a way that it functions as a suction aid and at the same time binds pollutant gas that escapes from the mask which will be treated in the laboratory.

### 2.3. Analysis of Samples with Atomic Absorption Spectrophotometers (AAS)

Gas analysis is carried out using AAS (Analytik Jena, Germany). The operating procedure for AAS is turned on, then the standard solution and sample are inserted into the test tube available on the AAS device, the computer AAS device is adjusted, the AAS flame and cathode lamp are turned on, and then the standard solution is sucked into Caitlin air, indicating that the reading measurement results must be zero by pressing the zero button. In a row, the standard solution was analyzed using AAS and the atomic absorption measurement results will be recorded and then calculated to get the concentration of gas captured in the sample range.

### 2.4. Data Analysis

Data analysis is used to decide the difference in decreasing toxic gases between controls by treatment types of mask material. Test statistics used analysis of variance to decide differences between treatments tested by t test. To determine the effectiveness of the gas absorb mask material, the post hoc LSD test was carried out. Conclusion is determined as follows; namely, if p value ≤ 0.05, then there is a significant difference between control and treatment.

## 3. Results 

**Figure 1:**
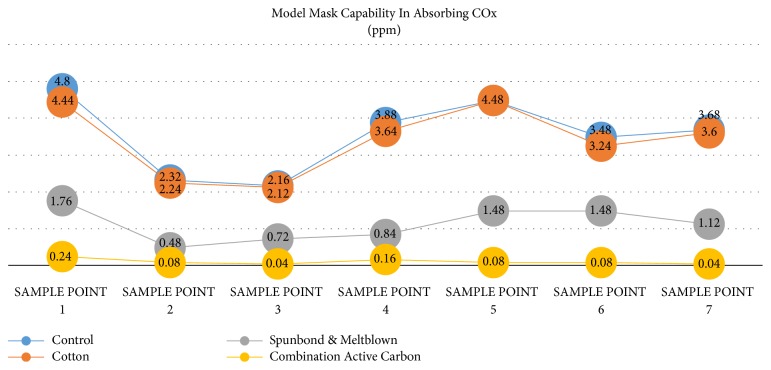
Various types of masks in absorbing COx.

The activated carbon combination mask model group has a higher ability than other mask models in absorbing COx. The average results of examination of exposure to COx exhaust emissions without using a mask (PPE)/control of 3.40 ppm (inhaled gas) are higher than using other masks, namely, cotton cloth material 3.39 n/mm3, spunbond, and meltblown 1.13 ppm and a mask of activated carbon combination material, spunbond, and meltblown of 0.10 ppm (see Figure 1).

Table 1 shows that the results of the analysis of variance test showed a significant difference between the decrease in COx exposure between controls and using masks (PPE) (p ≤ 0.001). The results of statistic test with LSD post hoc showed that there was no difference in effectiveness between control and cotton in filtering COx exposure (P = 0.71), whereas between the control with pound bound, control with activated carbon combination, spunbond, and meltblown, cotton cloth with spunbond and meltblown, and cotton cloth with activated carbon combination, spunbond, and meltblown, the result is a significant difference in effectiveness in lowering COx exposure between treatments (P-“0.001). There was a significant difference between the use of spunbond and meltblown masks and active carbon combination with spunbond and meltblown (P = 0.021).

**Figure 2:**
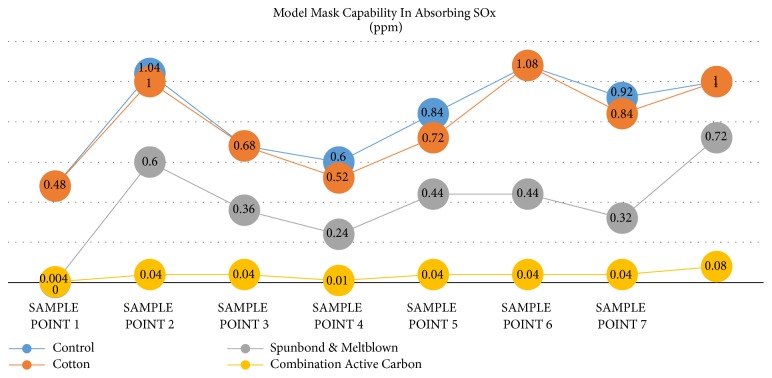
Various types of masks in absorbing SOx.

The results of examination of exposure to SOx exhaust emissions inhaled without using a mask (PPE) of 0.88 ppm are higher than using other masks, namely, cotton fabric material mask 0.84 n/mm3, spunbond, and meltblown 0.44 ppm and mask of activated carbon, spunbond, and meltblown 0.04 ppm (see Figure 2).

Table 2 shows that there is a significant difference SOx exposure between controls with treatment using masks (PPE) (P ≤ 0.001). The results of statistic test with LSD post hoc showed no difference in effectiveness between control and cotton in filtering COx exposure (P= 1.00), whereas between control and spunbond; control with activated carbon combination, spunbond, and meltblown; cotton cloth with spunbond and meltblown (p = 0.01); and cotton cloth with activated carbon combination, spunbond, and meltblown, the result is a significant difference in effectiveness in reducing COx exposure between treatments. Likewise, there is a difference between the use of spunbond and meltblown masks with activated carbon combinations with spunbond and meltblown (P≤ 0.001).

**Figure 3:**
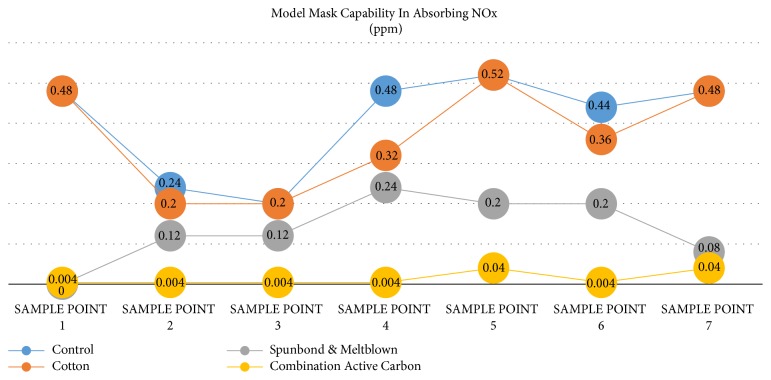
Various types of masks in absorbing NOx.

From the results of the average examination of NOx exhaust gas exposure without using a mask (PPE), the value is 0.48 ppm higher than using other masks, namely, cotton cloth material 0.37 ppm, spunbond, and meltblown and mask of activated carbon, spunbond, and meltblown is 0.014 ppm (see Figure 3).

Table 3 shows that the results of statistical tests have significant difference SOx exposure between controls with treatment using masks (PPE) (p ≤ 0.001). The results of statistic test with LSD post hoc showed that there was no difference in effectiveness between control and cotton cloth masks in decreasing COx exposure (P= 1.00). There is a difference between the control with spunbond (p = 0.041), there is a difference between the control with activated carbon, spunbond, and meltblown (P ≤ 0,001), and there is a difference between cotton fabric with spunbond and meltblown (P ≤ 0,001) and fabric cotton with activated carbon combination, spunbond, and meltblown (P ≤ 0.001). There was a significant difference between the use of spunbond and meltblown masks and active carbon combination with spunbond and meltblown in decreasing SOx gas exposure (P= 0.005).

## 4. Discussion

The high level of air pollution in Pontianak City is influenced by two main sources, namely, transportation and land fires. Pollutants COx, NOx, and SOx are the highest pollutants in the air, which come from man-made source [16]. SOx air pollutants are mostly from coal burning by 74%, industry by 22%, and transportation by 2% (Skinder et al., 2013). The case in Pontianak the highest pollution comes from sources of fire in agricultural or plantation land. The impact of COx exposure on public health, namely, the occurrence of COx poisoning due to the formation of carboxyhemoglobin (COHb) in the blood: the large COx activity compared to O2 to Hb causes the Hb function to carry oxygen throughout the body disrupted [1, 17].

Reduction of O2 in the blood (the body) will cause shortness of breath and can cause poisoning and SOx and NOx can stimulate the respiratory tract which causes irritation and inflammation. Ambient air pollution exposure, including COx, NOx, and SOx, in the long term will interfere with lung growth or development in the period of 10-18 years. The results of research conducted at the age of 8 to 18 years in California, USA, discussed the distance of residence closer to the freeway (motorway) is more at risk of lung growth deficit [1]. Long-term particulate matter/PM10 exposure to air pollution and nonaccidental mortality and toxic ratio (hazard ratio) cause of five specific deaths is higher than nonaccidental deaths, although statistically insignificant [18].

Control of toxic pollutants SOx, NOx, and COx is carried out in several ways, including nontechnical methods such as presenting environmental information, setting quality standards, industrial areas, and applying environmental impact assessment and technically such as changing industrial activity processes, installation of graphite deposition, and collector cyclone, while controlling the combination of technical and improving community discipline, namely, familiarizing people using personal protective equipment in the form of masks.

To prevent exposure to toxic substances in the air, either gas or particulate, several efforts are carried out, namely, technically, nontechnically, and administratively. Nontechnical control includes, for example, determining the area or industrial area, setting quality standards, and determining the environmental impact analysis. Meanwhile, technical control is the combustion engine to reduce the amount of pollutants formed during combustion, filters on the chimney, and the development of low-pollution power sources. Administrative controls include work rotation settings and mask use [19]. The use of personal protective equipment (masks) to prevent exposure to toxic materials in the air can be done, including the use of a nose and mouth mask (face mask) [20, 21]. The results showed that, of the three types of masks used, masks known as cotton cloth were not effective in reducing exposure to toxic substances COx, NOx, and SOx. Masks derived from cotton fabric have no different functions without using PPE in reducing pollutants COx, NOx, and SOx. This type of mask only works effectively to reduce dust particles measuring> 10 *μ* but cannot filter dust particulates smaller than that size and also prevent exposure to toxic gases.

While the mask model above has differences in filtering toxic gases, which are surgical mask, with spunbond and meltblown ingredients, some other types of masks are ordinary models, N95 and respirators. Regular model masks are used to cover the nose or mouth like a mask made from cotton cloth, handkerchief. Masks for hospitals, such as surgical masks, are for filtering dust and bacterial droplets and the type N95 which has the ability to filter bacteria and dust between 1 and 10 u to 95%. Respirator masks are usually used to provide material to filter and absorb substances contaminating toxic particles such as heavy metals [20, 21].

Activated carbon combination masks can cut toxic pollutants, especially characteristics of gas not absorbed or filtered on ordinary masks, such as COx, NOx, and SOx. The use of masks made from cotton cloth is not much different from the use of self-closing devices (masks). Effectiveness without masks for toxic gases is as follows: for COx by 89%, NOx 10%, and SOx 22%. The use of masks with cotton fabric is as follows: COx 85%, NOx 9%, and SOx 21%. The material is pound bound and meltblown is COx 27%, NOx 5%, and SOx 11%.

For this reason, it is necessary to design a mask that can filter or cut airborne pollution in or through the mask. The results of the study revealed that the mask combination with activated carbon and spunbond and meltblown had the ability to protect gas exposure. Combining activated carbon and spunbond and meltblown is very meaningful filtering (filtering) toxic gas material inhaled compared to other types of markers, namely, COx, SOx, and NOx (p <0.05).

The ability of the combination mask material made from spunbond meltblown has advantages and is a combination of several mask ingredients. Combination masks as one of the ingredients are spunbond and meltblown which is a mask commonly used for hospitals, as surgical masks. This model is for filtering dust and bacterial droplets. Besides that, the combination mask also adopted the N95 type which has the ability to filter bacteria and dust size between 1 and 10 u to 95% [20, 21], while other functions are as a respirator mask that can be used as an ingredient to filter and absorb substances contaminating toxic particles such as heavy metals or poisonous gases such as NOx, COx, and SOx. A mask is used as a personal protective equipment for workers, children, and the community. This is because a combination type mask besides having spunbond and meltblown ingredients also added granular activated carbon.

Masks using combination materials, especially absorption of activated carbon, showed a high ability compared to others, namely, being able to filter and absorb highly, so that toxic gas pollutants escaped very small, namely, COx 2%, NOx 0.3%, and SOx by 0.7%. Masks or PPE made from activated carbon and spunbond and meltblown are very meaningful filtering (filtering) and absorbing toxic gas material inhaled compared to other types of markers, namely, COx, SOx, and NOx (p <0.05). Masks other than spunbond and meltblown filter and absorb dust, bacteria, and particulates in the form of other harmful metals; this combination mask is made from activated carbon. Activated carbon is used in the form of a granule. Activated carbon absorbs gases that cannot be absorbed or filtered with ordinary masks or surgical masks and N95% masks. Use of activated carbon as a filter or absorbent to absorb gases including CO2 [22].

The ability of activated carbon to absorb COx, NOx, and SOx gas content is because the activated carbon has a relatively large volume of micropore and mesopore so that it has a large surface area [23]. Activated carbon is one type of absorbent where the structure of the carbon atom is a structure of amorphous carbon atoms, which are mostly composed of free carbon and have an inner surface, so that it has good absorption capacity. Coconut shell activated carbon around 50 mesh (0.297mm) has several active ingredients such as Fe 0.0353 mg, Zn 0.353, and Mn 0.0205 mg. Thus it is very possible be able to absorb harmful absorbed, namely, air poisonous gases such as COx, NOx, and SOx in sufficient quantities [24].

## 5. Conclusions and Recommendations

There is a number of effective mask combinations for activated carbon, spunbond, and meltblown in filtering toxic gases COx, SOx, and NOx (p <0.05). For this reason, clean prevention of gases can be overcome with gases which are active, bound, and melted.

## Figures and Tables

**Table 1:** Effectiveness of the use of masks and exposure to COx.

		n	mean (ppm)	CI 95%		*P* ^*a*^
				Min	Max	
COx not Adsorbed	Control	7	3,40	2,16	4,80	≤0.001 ^*∗*^

	Cotton	7	3,39	2,12	4,48	

	Spunbond & Meltblown	7	1,13	0,48	1,76	

	Combination Active Carbon, Spunbond & Meltblown	7	0,10	0,04	0.24	

^a^One-way ANOVA, *α* = 5%.

^*∗*^Significant (p ≤ 0,05).

Post hoc LSD: control vs cotton: P = 0.71; control vs spunbond & meltblown: P-“0,001; control vs combination active carbon, spunbond & meltblown: P-“0,001; cotton vs spunbond & meltblown: P-“0,001; cotton vs combination active carbon, spunbond & meltblown: P-“0,001; spunbond & meltblown vs combination active carbon, spunbond & meltblown: P = 0,021.

**Table 2:** Effectiveness of the use of masks and exposure to SOx.

		n	Mean	CI 95%		*P* ^*a*^
				Min	Max	
SOx not Adsorbed	Control	7	0.88	0.6	1,08	≤0.001 ^*∗*^

	Cotton	7	0.84	0.13	0.27	

	Spunbond & Meltblown	7	0.44	006	0.18	

	Combination Active Carbon, Spunbond & Meltblown	7	0.04	0.00	0.02	

^a^One-way ANOVA, *α* = 5%.

^*∗*^Significant (p ≤ 0,05).

Post hoc LSD: control vs cotton: P = 0.10; control vs spunbond & meltblown: P-“0,001; control vs combination active carbon, spunbond & meltblown: P = 0,01; cotton vs spunbond & meltblown: P = 0,01; cotton vs combination active carbon, spunbond & meltblown: P = 0,01; spunbond & meltblown vs combination active carbon, spunbond & meltblown: P-“ 0,001.

**Table 3:** Effectiveness of the use of masks and exposure to NOx.

		n	Mean (n/mm^3^)	CI95%		*P* ^*a*^
				Min	Max	
SOx not Adsorbed	Control	7	0.10	0.05	0.13	≤0.001 ^*∗*^

	Cotton	7	0.09	0.05	0.13	

	Spunbond & Meltblown	7	0.06	0.03	0.10	

	Combination Active Carbon, Spunbond & Meltblown	7	0.003	0.00	0.01	

^a^One-way ANOVA, *α* = 5%.

^*∗*^Significant (p ≤ 0,05).

Post hoc LSD: control vs cotton: P = 0.10; control vs spunbond & meltblown: P = 0,041; control vs combination active carbon, spunbond & meltblown: P = 0,01; cotton vs spunbond & meltblown: P-“0,001; cotton vs combination active carbon, spunbond & meltblown: P-“0,001; spunbond & meltblown vs combination active carbon, spunbond & meltblown: P= 0,05.

## Data Availability

The data used to support the findings of this study are included with in the article.
